# Homocysteine Induces Inflammation in Retina and Brain

**DOI:** 10.3390/biom10030393

**Published:** 2020-03-03

**Authors:** Nehal M. Elsherbiny, Isha Sharma, Dina Kira, Suhib Alhusban, Yara A. Samra, Ravirajsinh Jadeja, Pamela Martin, Mohamed Al-Shabrawey, Amany Tawfik

**Affiliations:** 1 Department of Oral Biology and Diagnostic Sciences, Dental College of Georgia, Augusta University, Augusta, GA 30912, USA; drnehal@hotmail.com (N.M.E.); ishashrikhand@yahoo.com (I.S.); dkira@augusta.edu (D.K.); salhusban@augusta.edu (S.A.); ysamra@augusta.edu (Y.A.S.); malshabrawey@augusta.edu (M.A.-S.); 2 James and Jean Culver Vision Discovery Institute, MCG, Augusta University, Augusta, GA 30912, USA; rjadeja@augusta.edu (R.J.); pmmartin@augusta.edu (P.M.); 3 Department of Biochemistry, Faculty of Pharmacy, Mansoura University, Mansoura 35516, Egypt; 4 Department of Biochemistry, Medical College of Georgia (MCG), Augusta University, Augusta, GA 30912, USA; 5 Department of Ophthalmology, MCG, Augusta University, Augusta, GA 30912, USA; 6 Department of Cellular Biology and Anatomy, Medical College of Georgia (MCG), Augusta University, Augusta, GA 30912, USA; 7 Department of Anatomy, Faculty of Medicine, Mansoura University, Mansoura 35516, Egypt

**Keywords:** homocysteine, inflammation, diabetic retinopathy, age-related macular degeneration, Alzheimer’s disease

## Abstract

Homocysteine (Hcy) is an amino acid that requires vitamins B_12_ and folic acid for its metabolism. Vitamins B_12_ and folic acid deficiencies lead to hyperhomocysteinemia (HHcy, elevated Hcy), which is linked to the development of diabetic retinopathy (DR), age-related macular degeneration (AMD), and Alzheimer’s disease (AD). The goal of the current study was to explore inflammation as an underlying mechanism of HHcy-induced pathology in age related diseases such as AMD, DR, and AD. Mice with HHcy due to a lack of the enzyme cystathionine-β-synthase (CBS) and wild-type mice were evaluated for microglia activation and inflammatory markers using immuno-fluorescence (IF). Tissue lysates isolated from the brain hippocampal area from mice with HHcy were evaluated for inflammatory cytokines using the multiplex assay. Human retinal endothelial cells, retinal pigment epithelial cells, and monocyte cell lines treated with/without Hcy were evaluated for inflammatory cytokines and NFκB activation using the multiplex assay, western blot analysis, and IF. HHcy induced inflammatory responses in mouse brain, retina, cultured retinal, and microglial cells. NFκB was activated and cytokine array analysis showed marked increase in pro-inflammatory cytokines and downregulation of anti-inflammatory cytokines. Therefore, elimination of excess Hcy or reduction of inflammation is a promising intervention for mitigating damage associated with HHcy in aging diseases such as DR, AMD, and AD.

## 1. Introduction

Recently, elevated levels of homocysteine (Hcy) has acquired much attention in clinical studies as a risk factor for many aging diseases such as diabetic retinopathy (DR) [1,2,3,4], age-related macular degeneration (AMD) [1,5,6,7] and Alzheimer’s disease (AD) [8,9,10,11]. Hcy is a non-proteinogenic thiol-containing amino acid endogenously liberated as a by-product of methionine transmethylation reactions. Hcy can be reprocessed into methionine by methylene-tetrahydrofolatereductase (MTHFR) or to cysteine by cystathionine-beta-synthase (CBS). Therefore, patients with mutations in MTHFR or CBS have elevated plasma Hcy levels, known as hyperhomocysteinemia (HHcy) [12]. In addition to CBS and MTHFR, proper metabolism of methionine and Hcy depends upon adequate supplies of folic acid and vitamin B_12_. Both vitamins play crucial roles in Hcy metabolism, they act as cofactors for enzyme methionine synthase to reprocess Hcy to methionine by the remethylation pathway using 5-methyltetrahydrofolate as a methyl donor [13]. Dietary deficiencies of folate and vitamin B_12_ are fundamental nutritional determinants of HHcy [14] that is exacerbated by high methionine intake. In the United States, the average diet is often low in folate and B_12_ relative to protein intake [15]. Nutritional deficiencies of vitamins B_12_ and folic acid are common in diabetic and aging populations and are linked to development of DR, AMD, and AD with concomitant elevation of Hcy [1,2,15,16,17,18].

Previously, we reported that HHcy disrupts inner and outer blood–retinal barriers (BRBs) [19,20], induces retinal ischemia and neovascularization [21,22], disrupts the retinal pigmented epithelium (RPE) structure and function with features of AMD [20], activates oxidative [19] and ER stresses [23], and induces epigenetic modifications [24]. Furthermore, we suggested Hcy as a prognostic marker for DR [25]. DR is associated with the breakdown of BRB. Moreover, RPE disruption is a cardinal feature of AMD. Emphasizing the fact that retina is part of the central nervous system (CNS), these deleterious impacts of HHcy highlight its cardinal role in the development and progression of aging retinal and brain diseases such as DR, AMD, and AD. The current study aims to explore inflammation as a potential underlying mechanism of HHcy-induced pathology in both the retina and brain with concomitant BRB [19,21] and blood–brain barrier (BBB) dysfunction [26,27].

Studies have identified a strong association between HHcy and inflammation in both human and experimental models [28,29,30]. Pathogenic levels of circulating Hcy are implicated in the induction of inflammatory determinants including the expression of adhesion molecules, leukocyte adhesion, endothelial dysfunction, oxidative stress, and reduced nitric oxide bioavailability [31]. In addition, high levels of Hcy were detectable in various inflammatory diseases such as inflammatory bowel disease [32], rheumatoid arthritis [33], and psoriasis [34]. HHcy has also been reported in clinical disorders associated with inflammatory conditions such as diabetes mellitus, cardiovascular diseases [35], and chronic kidney disease [36]. In ocular diseases, both Hcy and its metabolite Hcy-thiolactone show pro-inflammatory properties that induce optic nerve damage and visual dysfunction [18].

Microglia are the innate immune cells of the central nervous system (CNS). They play an important role in blood-vessel development, programmed cell death, debris phagocytosis, and activity-dependent synaptic pruning [37]. In response to injury, microglia rapidly migrate to the site of injury and undergo molecular and morphological changes associated with inflammation [38]. The impact of microglia activation following injury could be beneficial or detrimental, depending on the type of insult [39]. However, upregulation of neurotoxic or pro-inflammatory molecules by activated microglia is generally considered to be deleterious to neuronal survival [40,41]. Additionally, one pathway central to systemic inflammation is NF-κB, a transcription factor that regulates the transcription of various genes involved in inflammatory and immune responses. In unstimulated cells, NFκB is inactive in the cytoplasm, bound by IκB proteins. Activation of the NFκB pathway induces phosphorylation and degradation of IκBs, followed by nucleus translocation of free NF-κB where it promotes transcription of pro-inflammatory genes [42]. Activation of NF-κB signaling has been detected in the retina of DR [43] and AMD patients [44].

In vitro and in vivo experimental models of HHcy were used in the current study to understand the role of inflammation as a possible mechanism of HHcy-induced pathology in both the retina and brain. The in vivo experiments used mice lacking the gene encoding cystathionine-beta-synthase (CBS), a key enzyme needed to clear excess Hcy. Plasma Hcy levels range from moderate to severe, depending upon whether the mouse is heterozygous (*cbs*^+/−^) with one *cbs* copy or homozygous (*cbs*^−/−^), which has no copies of *cbs*. The *cbs*^+/−^ mice have about 4- to 7-fold increase in plasma homocysteine level and represent mild/moderate HHcy, and show a mild retinal phenotype and normal life span, while the *cbs*^−/−^ mice have about a 30-fold increase in plasma Hcy and represent severe HHcy with severe retinal phenotype, and a short life span of ~3 to 5 weeks. Our earlier published work showed that the *cbs*^−/−^ mice had severe changes in retinal vasculature in early age of life [21], while the *cbs*^+/−^ mice [22] have moderate changes in retinal vasculature, which become evident with aging. Both *cbs*^+/−^ and *cbs*^−/−^ mice were used in this study. In the in vitro studies, we used different types of retinal cells, which are implicated in retinal diseases. We used microglia as the main cell activated in inflammation in addition to HRECs and RPE, which play crucial roles in inner and outer BRB dysfunction in DR and AMD, respectively. Different concentrations of Hcy were also used to evaluate the effect of various levels of HHcy on the induction of inflammation in different cells and in different disease models, as reported in our previous studies [19,20,24].

Microglia activation and inflammatory markers were evaluated in vivo in the retinas and brains of HHcy mice that had cystathionine-β-synthase deficiency and in wild-type mice. Moreover, inflammatory responses of retinal epithelial cells, endothelial cells, and monocyte cell lines to HHcy were also investigated. Results of these studies suggest that targeting Hcy clearance or reduction of inflammation could provide a therapeutic avenue to mitigate retinal and brain diseases associated with HHcy.

## 2. Materials and Methods

### 2.1. Cell Culture

Human monocytes U937 cells (ATCC CRL-1593.2, Manassas, VA, USA, a generous gift from Doctor Gregory I. Liou, Department of Ophthalmology, Augusta University) were cultured at a density of 5 × 10^5^ cells/well in normal growth medium RPMI 1640 supplemented with glutamine 2 mM (Gibco, Invitrogen), 10% fetal bovine serum (Atlantic Biological, Norcross, GA, USA), and 1% penicillin streptomycin (Corning, Inc., NY, Catalog # 30-004-CI) at 37 °C. Cells were treated with L-homocysteine thiolactone hydrochloride (Hcy) (Sigma-Aldrich, Louis, MO) 20 µM for 24 h. The supernatants were collected and analyzed for pro-inflammatory cytokines by multiplex assay. Human primary retinal endothelial cells (HRECs) (Cell Systems Cooperation, Kirkland, WA) were cultured in EBM2 growth medium (Lonza, Walkersville, MD, Catalog #190860) supplemented with penicillin streptomycin 1% and fetal bovine serum (FBS) 5%. Human retinal pigmented epithelial cell line (ARPE-19) (American Type Culture Collection, ATCC, Manassas, VA, USA) at passage 6–15 was cultured in Dulbecco’s modified Eagle’s medium nutrient mixture F-12 (DMEM/F-12, Thermo-Scientific, Wyman, Massachusetts) containing penicillin/streptomycin 1% and fetal bovine serum 10%. At 80–90% confluency, the cells (HRECs and ARPE-19 cells and monocytes) were serum starved overnight and then Hcy treatment at (50 or 100 μM, choice of treatment doses according to our previous published work) or vehicle was applied for 24 h. Following treatment, the supernatant and/or the cells were harvested for further analyses.

### 2.2. Animal Studies

All animal experiments adhered to the recommendations accepted by the Association for Research in Vision and Ophthalmology (ARVO) Statement for the Use of Animals in Ophthalmic and Vision Research and were performed following our animal protocol approved by the Institute for Animal Care and Use Committee (IACUC) and Augusta University policies (protocol number AUP 2014-0683). Generation of mice deficient in cystathionine beta-synthase (*cbs*) was performed as previously described [45]. Pairs of *cbs*^+/−^ mice were purchased from Jackson Laboratories (B6.129P2-Cbstm1Unc/J; Jackson Laboratories, Bar Harbor, ME) and were bred to establish *cbs*^+/+^, *cbs*^+/−^, and *cbs*^−/−^ colonies. Mouse genotyping was performed as per the Jackson animal laboratory’s recommendations. Normal wild type mice (C57BL6/J) were injected with homocysteine thiolactone hydrochloride (Sigma-Aldrich, Louis, MO) to induce elevated HHcy or served as controls. Mice, 6–8 weeks of age, were injected with 1 μL homocysteine intravitreally as we previously described [20]. Briefly, homocysteine was dissolved in water to obtain (200 mM) stock solution that was diluted in 100 µL phosphate-buffered saline (PBS) solution to obtain a working solution. The *cbs^−/−^* mice were used at ages 3–5 weeks, which represent severe elevation of Hcy and have a shortened life span ~3–5 weeks, while the *cbs*^+/−^ represent mild/moderate elevation of Hcy level have a normal life span. 

### 2.3. Immunofluorescent Assessment of Mice Microglia Activation in Retina and Brain

Microglia activation was tested in brain frozen sections (hippocampus area) from *cbs*^+/+^, *cbs*^+/−^, and *cbs*^−/−^ mice, retinal frozen sections from wild type mice with Hcy intravitreal injection, and in retinal flat-mounts from *cbs*^+/+^, *cbs*^+/−^, and *cbs*^−/−^ mice, as described in our pervious publications [20,22]. For flat mounts, RPE flat-mount and inner retinal flat-mount were prepared. Eyes enucleated from *cbs*^+/+^, *cbs*^+/−^, and *cbs*^−/−^ mice, fixed in 4% paraformaldehyde overnight, were then transferred to PBS. RPE or inner retinal mounts were dissected, washed with PBS, then the mounts were incubated with Power Block (BioGenex, San Ramon, CA, USA) followed by incubation overnight at 4 °C with the primary antibodies rabbit polyclonal Iba1 (1:50, Wako Chemicals, Richmond, VA) and Griffonia simplicifolia isolectin-B4 (7 µL/mL, catalog number B-1105) Vector Labs (Burlingame, CA). The flat-mounts were subsequently washed three times in PBS–Triton X-100 followed by incubation with the appropriate secondary antibodies (Alexafluor and Texas red avidin, Invitrogen) for 1 h at 37 °C. Retinal flat-mount preparations were then washed and cover-slipped using Fluoroshield containing 4′,6-diamidino-2-phenylindole (DAPI) as a counter stain (Sigma Aldrich F6057, Saint Louis, MO, USA). Retinal flat mounts were visualized with anAxioplan-2 fluorescent microscope (Carl Zeiss, Göttingen, Germany) equipped with a high resolution microscope (HRM) camera (Carl Zeiss). Images were captured and processed using Zeiss Axiovision digital image processing software (version 4.8; Carl Zeiss). Samples were representative to at least three mice for each IF experiment.

### 2.4. Immunofluorescent Assessment of Inflammatory Markers in Retina and Brain

Inflammatory markers TNF-α and IL1β were assessed using immunofluorescence in frozen sections (eye and brain prepared from *cbs*^+/+^, *cbs*^+/−^, and *cbs*^−/−^ mice) as per our published method [19]. Briefly, frozen sections were fixed with 4% paraformaldehyde and blocked with Power Block, then incubated with primary antibody for TNF-α (1/200, ab1793) and IL1β (1/250, ab9722) from Abcam Corp. (Cambridge, MA) and biotinylated with GSL I-isolectin B4 (Vector Laboratories, Burlingame, Ca, Cat#: B-1205, 7μL/mL) for 3 h at 37 °C, followed by incubation with an appropriate secondary antibody (Alexafluor and Texas red avidin, Invitrogen). Next, sections were cover-slipped with Fluoroshield containing DAPI (Sigma-Aldrich) to label the nuclei. An Axioplan-2 fluorescent microscope (Carl Zeiss, Göttingen, Germany) equipped with a high resolution microscope (HRM) camera was used to capture images using Zeiss Axiovision digital image processing software (version 4.8). Samples were representative to at least three mice for each IF experiment.

### 2.5. Western Blot Analysis to Detect Cytoplasmic and Nuclear Levels of NF-κB

Following Hcy treatment, nuclear extracts of HRECs and ARPE-19 were prepared using a nuclear extraction kit purchased from Abcam (ab113474) (Abcam Inc., Cambridge, MA, USA) to detect nuclear levels of NF-κB. Both nuclear extracts and cytoplasmic extracts were subjected to gel electrophoresis on sodium dodecyl sulfate-polyacrylamide gel (SDS-PAGE). The protein was blotted to nitrocellulose membranes, which were further blocked using 5% milk solution and then incubated with the NF-κB antibody (1:300, Cell Signaling, Danvers, MA, USA) overnight at 4 °C. Membranes were then re-probed with histone deacetylase (HDAC) (Abcam Inc., Cambridge, MA, USA) as the loading control for nuclear extracts, and actin (Abcam, ab5694, Cambridge, MA, USA) as the loading control for cytoplasmic extracts. Blots were then incubated with an appropriate peroxidase-conjugated secondary antibody. An enhanced chemiluminescence (ECL) western blot detection system (Thermo Scientific) was used for visualization of protein bands and ImageJ software was used to determine the optical density of the bands. Data are presented as ratio of nuclear to cytoplasmic NF-κB. Samples were representative to at least three mice for each experiment.

### 2.6. Establishment of Primary Retinal Pigment Epithelium (RPE) Cell Cultures

Age-matched (~3 week old) wild type and cbs^+/−^ mice were used for the preparation of primary RPE cell cultures as previously published [46]. Briefly, after enucleation, mouse eyes were rinsed in 5% povidone-iodine solution, then rinsed with sterile Hank’s Balanced Salt Solution (HBSS). Eyes were then sited in cold RPE cell culture medium (DMEM:F12), which is supplemented with 25% fetal bovine serum, 0.1 mg/mL gentamicin, 100 U/mL penicillin, and 100 µg/mL streptomycin. Then, eyes were incubated in HBSS containing 19.5 U/mL collagenase and 38 U/mL testicular hyaluronidase for 40 min at 37 °C, followed by incubation in HBSS containing 0.1% trypsin (pH 8) for 50 min at 37 °C. To separate RPE from the neural retina, eyes were dissected and isolated RPE cells were collected in a 15 mL centrifuge tube and centrifuged at 1228× *g* for 10 min (Thermo Medilite Centrifuge, Thermo Scientific, Waltham, MA), followed by suspension and culture of RPE cells in RPE cell media at 37 °C.

### 2.7. ELISA (Enzyme-Linked Immunosorbent Assay) to Detect NF-κB p65

An ELISA kit was used to evaluate the effect of elevated Hcy on NFκB activation by measuring the level of NFκB p65 in HRECs and ARPE-19 treated with Hcy (50 and 100 μM) for 24 h. After treatment, media was removed, followed by washing twice by PBS. Then, the cells were solubilized by using cell extraction buffer and NFκB p65 was measured in these cell lysates using ELISA (ab176648 ELISA Kit, Abcam, Cambridge, MA, United States). Absorbance was measured by a plate reader at 450 nm.

### 2.8. Analysis of Inflammatory Cytokines

Two methods were used to assess the inflammatory cytokines. 

#### 2.8.1. Human Cytokine Array

For detection and quantitation of pro-inflammatory cytokines and anti-inflammatory cytokine produced by HRECs and ARPE-19, a cytokine array kit was used as described previously [47]. Briefly, HRECs and ARPE19 cells were treated with or without homocysteine thiolactone (50 µM and 100 µM, respectively) for 24 h. Cell culture supernatant was collected and assayed for secreted inflammatory cytokines using the Human Cytokine Array C5 platform (RayBiotech, Norcross, GA, USA). Three replicates per sample and two replicates per cytokine were performed. These arrays produce protein expression pattern profiles for 62 different cytokines. Membranes were blocked with 1X assay diluent followed by incubation overnight with cell culture supernatant at 4 °C. Membranes were incubated with biotin-labeled anti-cytokines followed by incubation with avidin-horseradish peroxidase (1:1000). Membranes were developed using enhanced chemiluminescence (Thermo Scientific, Pittsburgh, PA, USA) and detected on HyBlot CL™ autoradiography film (Denville Scientific, South Plainfield, NJ, USA). Spots (dots) on membranes were quantified using ImageJ (Version 1.45; NIH, Bethesda, MD, USA) to determine the integrated signal density for each cytokine and was normalized against the internal standards provided with the array. Fold-differences in cytokine secretion were reported for treatment groups relative to controls. 

#### 2.8.2. Multiplex Cytokine Assay

Simultaneous quantitation of pro-inflammatory cytokines and anti-inflammatory cytokines produced by HRECs, ARPE-19, and the human monocyte cell line U93 were quantified for cells treated in the presence and absence of Hcy. The Bio-Plex human cytokine assay (Bio-Rad, Hercules, CA) was used for this assay, according to the manufacturer’s instructions. Briefly, serial dilutions of standard solutions of known cytokines were used to generate corresponding standard curves. In a 96-well filtration plate, premixed beads coated with target capture antibodies were added to each well, followed by washing twice with Bio-Plex wash buffer. Premixed samples (supernatant from *cbs*^+/−^ hippocampal brain tissue, Hcy-treated human monocyte cell line U937, HRECs, and ARPE-19 cells) or standards were added (three replicates of samples and two replicates for each cytokine). The plate was shaken for 30 s and then incubated with shaking at 300 rpm for 30 min at room temperature. Following incubation, the media was aspirated from the wells, then the wells were washed and detection antibodies were added with shaking for 10 min at room temperature. The wells were washed three times and Bio-Plex assay buffer was added. Reading was performed using the Bio-Plex suspension array system (Bio-Rad, Hercules, CA) and Bio-Plex Manager™ software version 3.0 (Bio-Rad, Hercules, CA) was used for data analysis.

### 2.9. Statistical Analysis

Comparisons between the experimental groups were done using the student’s two-tailed t test or one way analysis of variance (ANOVA), with a post hoc Tukey’s test. *p* value < 0.05 was considered statistically significant. Results are expressed as mean ± SD. 

## 3. Results

### 3.1. Hcy Induces Inflammatory Response in Mouse Retina, Brain, and Cultured Human Monocytes

**Figure 1:**
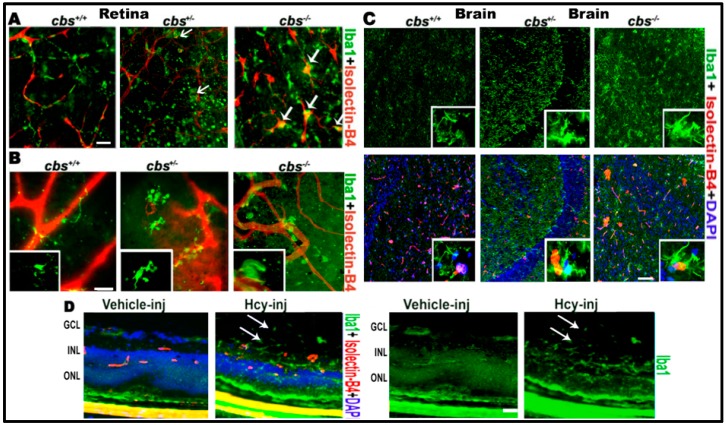
Hcy activates microglia in retina and brain. Microglia activation in retinal flat-mounts of *cbs*^−/−^, *cbs*^+/−^ mice and brains of *cbs*^−/−^ and *cbs*^+/−^ mice (**A**) RPE flat-mount (**B**) inner retinal flat-mount, and (**C**) brain frozen sections compared to *cbs*^+/+^ mice. Iba1 microglia marker (green) shows quiescent ramified microglia in *cbs*^+/+^ and rod-like microglia devoid of branching processes stains for both Iba1 and isolectin-B4 (white arrows, yellow stain) in the *cbs*^−/−^ and cbs^+/^^–^ retinas and brains indicating microglia activation. In addition, Hcy activated microglia (green) in the retinal section from normal wild type mice injected intravitreal with Hcy (**D**). Samples were representative to at least three mice for each immuno-fluorescence (IF) experiment. Scale bars: 20, 20, 50, and 20 μm, respectively. Abbreviations: GCL = ganglion cell layer, INL = inner nuclear layer, ONL = outer nuclear layer.

First, we examined the role of HHcy in microglial activation in the mouse central nervous system (CNS) (retina and brain). In the retina, we used layer specific retinal flat mounts and frozen sections to examine microglia activation. Immuno-localization of microglia in retina using microglia marker Iba1 and blood vessel marker isolectin-B4 (ISB) demonstrated microglia activation in RPE flat-mounts of *cbs*^−/−^ (three weeks old) and *cbs*^+/−^ mice (one year old) (Figure 1A) and in the inner retinal flat mount of *cbs*^−/−^ (three weeks old) and *cbs*^+/−^ mice (one year old) (Figure 1B). Normal retinal flat-mount preparations from *cbs*^+/+^ mice showed quiescent ramified microglia, while *cbs*^−/−^ and *cbs*^+/−^ retinas demonstrated rod-like microglia devoid of branching processes, indicating microglia activation in HHcy mice (indicated by white arrows). These data are consistent with our previous published retinal data [21,22] showing *cbs*^−/−^ mice (severe HHcy) have marked changes in retina compared to *cbs*^+/−^ mice (mild/moderate HHcy), which in turn showed marked changes compared to *cbs*^+/+^ mice. Microglia activation was obvious in *cbs*^−/−^ mice at a very young age (three weeks) while age matched *cbs*^+/−^ mice showed milder activation of microglia, which became more evident with increased age. Furthermore, we wanted to know whether impacts of elevated Hcy and induction of inflammation was limited to the retina or also extended to the brain. To answer this question, we tested the microglia activation in the brain and found similar activation of microglia in the brain of age matched *cbs^−/−^* and *cbs*^+/−^ mice (three weeks old) compared to wild type *cbs*^+/+^ mice (Figure 1C). Interestingly, microglia activation was observed in both *cbs*^−/−^ and *cbs*^+/−^ mice brains with an abundance of Iba1 positive activated microglia in the hippocampal area as early as three weeks of age. To verify the contribution of Hcy to the microglial activation observed in the *cbs*^−/−^ and *cbs*^+/−^ mouse retina, we next examined microglial activation in wild-type retinas (6–8 weeks old) 48 h after intravitreal injection of Hcy or PBS solution (vehicle) (Figure 1D) Immuno-localization of microglia in retinal frozen sections using microglia marker Iba1 showed activation of microglia, which was also extended to the vitreous (arrows).

### 3.2. HHcy Activated Nuclear Translocation of Transcription Factor NF-κB in Cultured Retinal Cells and Isolated cbs^+/−^ Mice Primary RPE Cells

**Figure 2:**
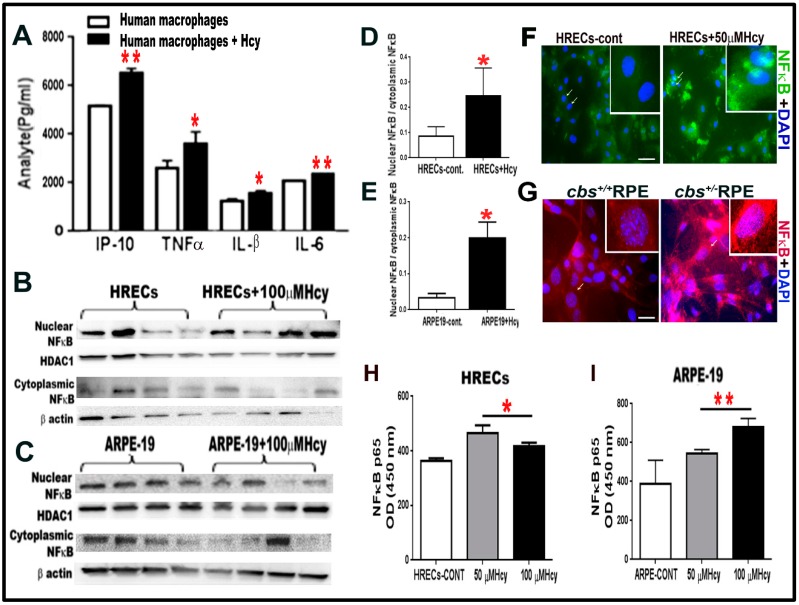
(**A**) Multiplex assay for human monocyte cell line U937 treated with and without Hcy 20 μM for 24 h revealed significant increase in pro-inflammatory cytokines (IP-10, TNFα, IL-B, and IL-6). (**B**–**E**). HHcy activates NF-κB transcription factor in cultured HRECs and ARPE-19 cells. Western blot analysis for HRECs (**B**,**D**) and ARPE-19 cells (**C**,**E**) treated with 100 µM Hcy, showing significant increase in the NF-κB nuclear to cytoplasmic ratio compared to the control untreated cells. NF-κB is a transcription factor, which controls the transcription of DNA and cytokine production. (**F**) IF staining for NF-κB, showed that Hcy activated and translocated NF-κB from the cytoplasmic to the nuclear side in HRECs treated with Hcy (green). (**G**) Primary RPE cells isolated from *cbs*^+/−^ mice retina (red). (**H**–**I**) ELISA measurement of NFκB (P65) in HRECs (H) and ARPE-19 (I) treated with and without Hcy (50 μM and 100 μM) for 24 h. Samples were representative to at least three mice for each experiment. Data are expressed as mean ± SD. (* *p* < 0.05, ** *p* < 0.01). Scale bar: 50 and 20 μm, respectively.

Next, we asked whether Hcy induced inflammation was limited to the direct activation of microglial cells or if it also occurred in other retinal cell types. For these experiments, the human monocyte cell line U937, which has the potential of differentiation into macrophages (pre-microglia), was used. U937 cells were treated with and without homocysteine thiolactone 20 μM for 24 h and showed significant increase in pro-inflammatory cytokines IP-10, TNFα, IL-1β, and IL6 following Hcy treatment (Figure 2A). Then, we asked whether HRECs and RPE upregulated inflammation directly in response to Hcy independent of microglia induced inflammation. To do this, we examined the activation of NF-kB, a transcription factor that controls the expression of various inflammatory cytokines. Nuclear and cytoplasmic levels of NF-κB, were measured by western blot analysis (Figure 2B,E) in HRECs and ARPE-19 treated with 100 µM homocysteine thiolactone for 24 h. Results showed significantly higher ratios of nuclear/cytoplasmic NF-κB in Hcy treated cells compared to vehicle treated cells, indicating that Hcy-induced NF-κB activation and nuclear translocation. In addition, IF staining for NF-κB showed activation and translocation of NF-κB from the cytoplasmic to the nuclear compartments in HRECs treated with Hcy 50 µM for 24 h (Figure 2F) and RPE cells isolated from the *cbs^+/−^* eyes (Figure 2G).

Additionally, an ELISA kit, which measures NF-κB p65 in cell lysate, was used to confirm the WB and IF data of NF-κB activation (Figure 2H, I). The ELISA data showed significant elevation of activated form of NF-κB (p65) in both HRECs (Figure 2H) and ARPE cell (Figure 2I) treated with Hcy (50 µM and 100 µM for 24 h.) compared to control untreated cells.

### 3.3. HHcy Increased IL1β and TNF-α Expression in Retina and Hippocampus in cbs^−/−^, cbs^+/−^ and cbs^+/+^ Mice Brain

**Figure 3:**
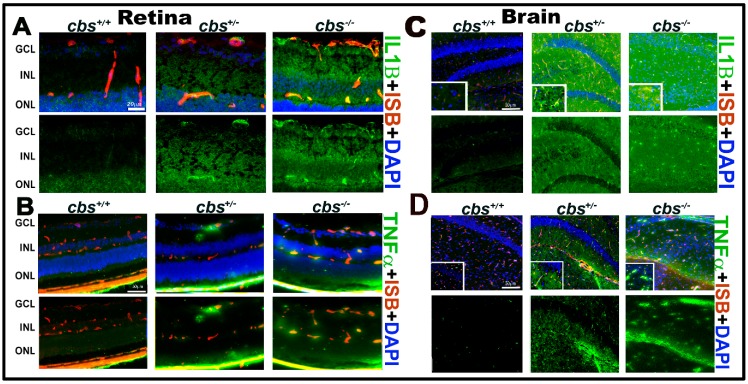
HHcy activates IL-1β in retina and TNF-α in retina and hippocampus area of the *cbs* mice brain. (**A**) IF staining for retinal frozen section from the *cbs*^−/−^, *cbs*^+/−^, and *cbs*^+/+^ mice for IL1β (green); (**B**) TNFα (green, retina); (**C**) IL1β (green, brain); (**D**) TNFα (green, brain), and isolectin-B4 (ISB) for the blood vessels (red, **A**–**D**) showing the activation of retinal IL1β and TNFα in the retinal and brain by HHcy. Samples were representative to at least three mice for each experiment. Scale bar: 20, 50, 50, and 50 μm, respectively. Abbreviations: GCL = ganglion cell layer, INL = inner nuclear layer, ONL = outer nuclear layer.

Based on the detection of cytokines secreted by Hcy treated U937 cells, we next investigated IL-1B and TNF-α expression by IF staining of frozen sections from *cbs*^−/−^, *cbs*^+/−^, and *cbs*^+/+^ mouse retinas (Figure 3A,B) and brain hippocampal areas (Figure 3C,D). Retinal staining showed marked increase in the expression of the inflammatory factors IL1β (green, Figure 3A) in *cbs*^+/−^ and *cbs*^−/−^ mice retina and TNF-α (green, Figure 3B) compared to *cbs*^+/+^ mice. Moreover, brain staining showed marked increase in IL1β (green, Figure 3C) and TNF-α (green, Figure 3D) levels in the hippocampal area in the *cbs*^+/−^ and *cbs*^−/−^ mice brain compared to *cbs*^+/+^ mice (mice age ~3 weeks old). 

### 3.4. Cytokine Array Assay for Cytokines Secreted from Human ARPE-19 and HRECS Cells Treated with Hcy

**Figure 4:**
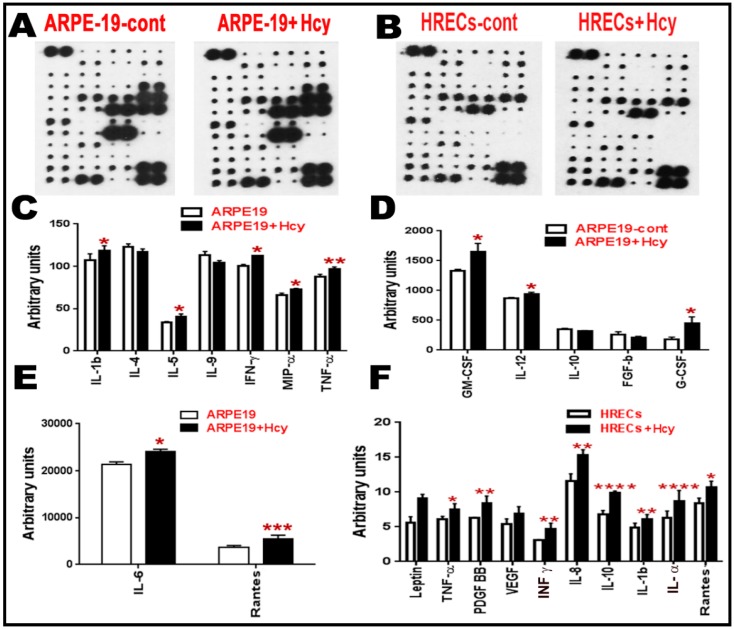
Cytokine array analysis for retinal cells treated with Hcy for 24 h. (**A**–**F**) Cytokine array analysis of ARPE cells and HRECs treated with Hcy for 24 h. (**A**,**B**) Membranes showing the change in the inflammatory cytokines. The intensities of the spot were quantified densitometrically and compared to the density of the internal standards, yielding an integrated density value (IDV) for each cytokine. (**C**–**E**) Analysis showing significant increase of pro-inflammatory cytokines (IL-1β, IL5, IFN-γ, MIP-α, TNF-α, GM-CSF, IL-12, G-CSF, IL6, and Rantes) and downregulation of anti-inflammatory cytokines (IL-4 and IL10) when ARPE-19 cells were treated with 100 µM Hcy for 24 h. (**F**) HRECs treated with Hcy-50 µM for 24 h showed significant increase in pro-inflammatory and pro-angiogenic cytokines (TNF-α, PDGF BB, IFN-γ, IL8, IL10, IL-1 β, IL-α, and Rantes) and a marked increase in leptin and VEGF. Data are expressed as mean ± SD. (* *p* < 0.05, ** *p* < 0.01, *** *p* < 0.001, **** *p* < 0.0001).

Given the finding that NFkB is activated in ARPE-19 and HRECs cells, we next investigated the downstream expression of various inflammatory cytokines in these two cell types. Cytokines are secreted cell–cell signaling proteins that play important roles in inflammation, angiogenesis, innate immunity, apoptosis, and cell differentiation and growth. Quantitative data for cytokines released in the conditioned media of ARPE-19 and HRECS cells with/without homocysteine thiolactone treatment (Figure 4A–F) were evaluated. The intensities of the spots representing cytokines on the detection membrane (Figure 4A,B) were quantified densitometrically and compared to the density of the internal standards, yielding an integrated density value (IDV) for each cytokine. Quantitative data for cytokines released in the conditioned media of ARPE-19 cells and HRECS in response to 100 μM and 50 μM homocysteine thiolactone appear in (Figure 4C–F), respectively. Out of the 80 array cytokines, cytokine array analysis showed significant increases in pro-inflammatory cytokines (IL-1β, IL-5, IFN-γ, MIP-α, TNF-α, GM-CSF, IL-12, G-CSF, IL-6, and Rantes) and downregulation of anti-inflammatory cytokines (IL-4, IL-10) in ARPE-19 cells treated with 100 μM homocysteine thiolactone for 24 h (Figure 4C–E). Moreover, HRECs treated with Hcy-50 μM for 24 h showed a marked increase in pro-inflammatory and pro-angiogenic cytokines (Leptin, TNF-α, pigment epithelium-derived factor (PEDF), vascular endothelial growth factor (VEGF), interferon-gamma (IFN-γ), IL-8, IL-10 IL-1β, IL-α, and Rantes) (Figure 4F).

### 3.5. Multiplex Assay for Cytokines Secreted from Human ARPE-19 and HRECs Cells Treated with Hcy and cbs^+/−^ Mice Brain 

**Figure 5:**
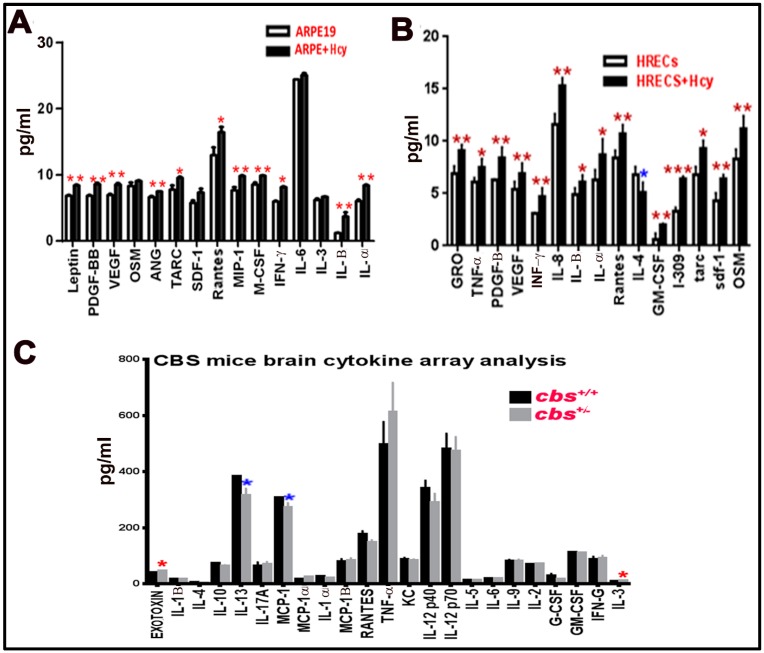
Multiplex assay for cytokines secreted from retinal cells treated with Hcy and *cbs*^+/−^ mice brain. Multiplex cytokine assay analysis from conditioned media of HRECS and ARPE-19 cells in response to 50 µM and 100 µM Hcy, respectively. (**A**,**B**) Showed and confirmed the increased of the pro-inflammatory cytokines and downregulation of the anti-inflammatory cytokine (IL4), while brain cytokine array from the hippocampal area of the *cbs*^+/−^ mice brain (**C**) showed a significant decrease in IL13, MCP-1, and increase in Exotoxin and IL3. Three replicates per sample and two replicates per cytokine were performed (* *p* < 0.05, ** *p* < 0.01, *** *p* < 0.001). Data are expressed as mean ±SD.

Multiplex assay was used as a second method to measure and confirm the elevation of inflammatory cytokine by HHcy. In the retina, we wanted to test the effect of Hcy on HRECs and RPE cells separately rather than the whole retina (as HRECs are mainly affected in inner BRB dysfunction, which is more related to DR, and RPE is mainly affected in the outer BRB dysfunction, which is more related to AMD). Secreted cytokines were elevated in retinal cells treated with homocysteine thiolactone (Figure 5A,B) and in *cbs*^+/−^ mice (12 weeks old) brain hippocampus area compared to *cbs*^+/+^ (Figure 5C). The retinal cells multiplex array data were consistent with the retinal cell membrane cytokine assay data. In addition, a multiplex array of HRECs treated with Hcy showed significant decrease of anti-inflammatory cytokine IL-4 (Figure 5B). The brain cytokine array data showed a significant decrease in the anti-inflammatory cytokine IL13 and MCP-1 and increase of pro-inflammatory cytokine IL3 and Exotoxin.

## 4. Discussion

The present study demonstrates that activation of inflammatory signaling is implicated in HHcy-induced BRB dysfunction and hippocampus atrophy. Our data from hyperhomocysteinemic mouse model (*cbs*^+/−^ and *cbs*^−/−^ or wild type mice that received intravitreal injection of Hcy) showed activation of microglial cells and increased expression of pro-inflammatory cytokines such as IL-1B and TNFα. Similar results were obtained from our in vitro studies where microglia were subjected to excess Hcy. Furthermore, excess Hcy upregulated a wide variety of pro-inflammatory cytokines, while downregulated anti-inflammatory cytokines in the key cells that constitute BRB, HRECS, and RPE. Our results are consistent with increasing evidence showing that systemic inflammation, along with local retinal or brain inflammation, can play a significant role in the development and progression of DR, AMD, and AD [48,49,50,51,52].

Rhodehouse et al. and Beard et al. [53,54] reported blood–brain barrier dysfunction in the *cbs*^+/−^ mice and we reported deleterious effects of elevated Hcy on inner and outer BRB integrity both in vivo using the *cbs*^+/−^ and *cbs*^−/−^ mice and in vitro using cultured retinal cells [20,22]. However, the underlying mechanisms of HHcy–induced barrier dysfunction need further investigation. Recently, we reported endoplasmic reticulum (ER) stress [23], oxidative stress [19], and epigenetic modification [24] as possible mechanisms, however, major questions remain regarding the role of inflammation in this process.

Retina is a highly sensitive and metabolic neurovascular tissue. Accumulation of noxious factors, prolonged exposure to stress, and/or dysfunction drive detrimental chronic unbalanced and uncontrolled inflammatory responses in retinal tissue [55]. Additionally, activation of inflammation in the CNS is believed to play an important role in the pathogenesis of neurodegenerative diseases such as AD. The inflammatory response is believed to be mediated by the activated microglia with subsequent release of potentially cytotoxic molecules such as pro-inflammatory cytokines, reactive oxygen intermediates, and proteinases, leading to neuronal cell death [48].

Microglia activation and the release of inflammatory cytokines have been reported to initiate BRB breakdown in DR [56], ocular autoimmunity [57], and retinal degenerative diseases [58]. Consistent with the established role of microglia in the induction of inflammation, our data showed evidence of microglia activation in both the inner and outer retina as well as brain hippocampus, as shown by microglia morphological transformation to an amoeboid shape with larger cell bodies and shorter thicker processes, suggesting enhanced immunoreactivity, increased phagocytic activity, and the production of pro-inflammatory mediators [59]. Given the known involvement of both inflammation and Hcy in DR, AMD, and AD, it is important to understand the role of HHcy in CNS inflammation. Previous studies have reported that Hcy induced vascular inflammation was mediated via activation of NF-κB in vascular smooth-muscle cells [60]. Furthermore, Hcy was reported to contribute to the induction of cerebral ischemia through the induction of cerebral microglia activation [61] and upregulation of pro-inflammatory cytokine production [62].

Consistent with these reports, our WB, IF, and ELISA data showed that different concentrations of elevated Hcy (50 µM, representing moderate HHcy and 100 µM, representing severe HHcy) induced the activation of NF-κB in cells that play a crucial role in maintaining intact BRB (HRECs and ARPE cells). This inflammatory effect elicited by Hcy was further confirmed by the cytokine array, which revealed increased production of pro-inflammatory and pro-angiogenic factors and decreased levels of anti-inflammatory cytokines in HRECs and RPE cells subjected to Hcy treatment. Our results support a role of the inflammatory milieu triggered by excess Hcy in both the inner and outer BRB. Furthermore, the Hcy-induced inflammation was evaluated in vivo, where sections from both the retina and brain of *cbs* mice showed microglia activation and marked immunostaining of NFκB and pro-inflammatory cytokines TNF-α and IL1β.

HHcy has also been implicated in AD, which affects the hippocampus area. In AD, the hippocampus area is atrophied, which explains the disorientation and memory loss that characterize AD patients. Interestingly, we also noticed atrophy in the hippocampus area of the *cbs*^−/−^ mice brain (3–5 weeks of age). Various investigations demonstrated that sustained inflammatory response is evident in the brain of AD patients [63]. These inflammatory events are thought to exacerbate β-amyloid and tau pathologies, which further potentiate the severity of AD [64] and cause the destruction of neurons and induces the loss of synaptic proteins, leading to cognitive deficits [65]. Zhang et al. reported a correlation between HHcy and characteristic AD pathological features including hippocampus β-amyloid (Aβ) deposition [66] and tau hyperphosphorylation [67]. Moreover, clinical studies demonstrated an association between HHcy and cognitive decline as well as cognitive Alzheimer’s impairment [68]. HHcy in the elderly population (≥60 years) resulted in doubling the risk of AD compared to those with lower blood Hcy level [69]. However, the exact molecular mechanisms have not been fully defined yet [70].

Herein, we showed that different levels of elevated Hcy were able to trigger inflammatory response in brain of the HHcy mice as evident by microglia activation and increased levels of inflammatory markers in hippocampus of the *cbs*^−/−^ and *cbs*^+/−^ mice (representing severe and mild/moderate HHcy, respectively). In the current study, the brain cytokine array data showed significant decrease in IL13 and MCP and increase in IL3 and Exotoxin by HHcy. IL13 is a well-known anti-inflammatory cytokine that exhibits anti-inflammatory activities by inhibiting the production of inflammatory cytokines [71]. IL3 was reported to be one of the 5-protein biomarker molecular signature for predicting Alzheimer’s disease [72]. However, exotoxins are generally complex soluble polypeptides produced on the inside of pathogenic bacteria as part of their normal growth and metabolism, and these are typically excreted by living cells or released during bacterial cell lysis into the surrounding medium. Interestingly, bacterial lipopolysaccharide (LPS) was found in brain lysates from the hippocampus and superior temporal lobe neocortex of Alzheimer’s disease brains, which were reported to be pathogenic and highly detrimental to the homeostatic function of neurons in the CNS [73].

In conclusion, our data suggest that HHcy-induced inflammation could play a role in BRB and BBB dysfunction and the pathogenesis of DR, AMD, and AD. Thus, the elimination of excess homocysteine could be a potential therapeutic intervention in DR, AMD, and AD. This could be through adequate nutritional vitamin supplementation of folic acid and vitamin B-12, especially in the aging and diabetic population. Furthermore, genetic or pharmacological manipulation of the CBS gene could be an innovative approach to clear excess homocysteine.
